# Stochastic attractor models of visual working memory

**DOI:** 10.1371/journal.pone.0301039

**Published:** 2024-04-03

**Authors:** W. Penny

**Affiliations:** School of Psychology, University East Anglia, Norwich, United Kingdom; AGH University of Science and Technology Faculty of Physics and Applied Computer Science: Akademia Gorniczo-Hutnicza im Stanislawa Staszica w Krakowie Wydzial Fizyki i Informatyki Stosowanej, POLAND

## Abstract

This paper investigates models of working memory in which memory traces evolve according to stochastic attractor dynamics. These models have previously been shown to account for response-biases that are manifest across multiple trials of a visual working memory task. Here we adapt this approach by making the stable fixed points correspond to the multiple items to be remembered within a single-trial, in accordance with standard dynamical perspectives of memory, and find evidence that this multi-item model can provide a better account of behavioural data from continuous-report tasks. Additionally, the multi-item model proposes a simple mechanism by which swap-errors arise: memory traces diffuse away from their initial state and are captured by the attractors of other items. Swap-error curves reveal the evolution of this process as a continuous function of time throughout the maintenance interval and can be inferred from experimental data. Consistent with previous findings, we find that empirical memory performance is not well characterised by a purely-diffusive process but rather by a stochastic process that also embodies error-correcting dynamics.

## 1 Introduction

Working Memory (WM) [1] and short-term memory [2] refer to our ability to maintain information over time in the absence of direct sensory input. This allows us to decouple behaviour from our immediate world and underlies a host of functions from attention, to executive function, planning and problem solving. Moreover, deficits in working memory are implicated in a host of neurological disorders including Parkinson’s and Alzheimer’s [3].

A well known feature of working memory is that it has a limited capacity, limited perhaps in the discrete number of items that can be remembered [4] or the precision with which items can be recalled [5, 6]. Errors in short-term memory are thought to be due, in part, to noise in the underlying neuronal representations from the individual failures of synapses or the stochastic spiking of single neurons. This noise can cause memory representations to diffuse away from their initial states leading to memory errors [7, 8].

Theoretical and empirical work has shown that working memories can be stored in continuous “ring” attractors and that this form of storage tends to accumulate errors over time in a manner commensurate with a stochastic diffusion process [9]. A further body of work, however, shows that errors accumulated by diffusion processes can be corrected through the use of “discrete” attractors, wherein memories are stored as stable fixed points of a dynamical system [10, 11]. Here, the fate of a memory is governed by the balance of two forces, a stochastic force that causes memory traces to randomly diffuse over time and a deterministic force that directs traces toward attractor states. In this view, it is this second deterministic force that provides memory systems with their robustness and error-correcting abilities.

In recent work Panichello et al. [12] developed a model of stochastic attractor dynamics and fitted it to data from a continuous-report visual working memory task. A key feature of their work is that they were able to identify stable fixed points that corresponded to biases in participant’s responses across a set of trials. For example, instead of reporting the precise variant of blue, participants tended to report an “archetypal” blue and this tendency increased with maintenance interval length, reflecting convergence towards a stable state. We refer to their approach as the “Response-Bias” model.

Generically, the bias-variance decomposition [13] shows that the errors of any system can be decomposed into those caused by “bias” and those caused by “variance”. One way of decreasing the overall error is to introduce a small (correct) bias which will have the effect of reducing the variance. The Response Bias model is motivated by this perspective, and the empirical finding that participant responses cluster around archetypal values over trials [12, 14, 15].

In this paper we use a similar mathematical formalism [12] but propose assigning stable fixed points to the multiple items that are to be remembered on each trial. This is consistent with the original construct of discrete attractor dynamics [16, 17] and circuit-level implementations using, for example, Dynamic Field Theory [18]. We show that this “Multi-Item” model can provide a better account of data from continuous-report visual working memory tasks.

### 1.1 Continuous-report tasks

**Fig 1:**
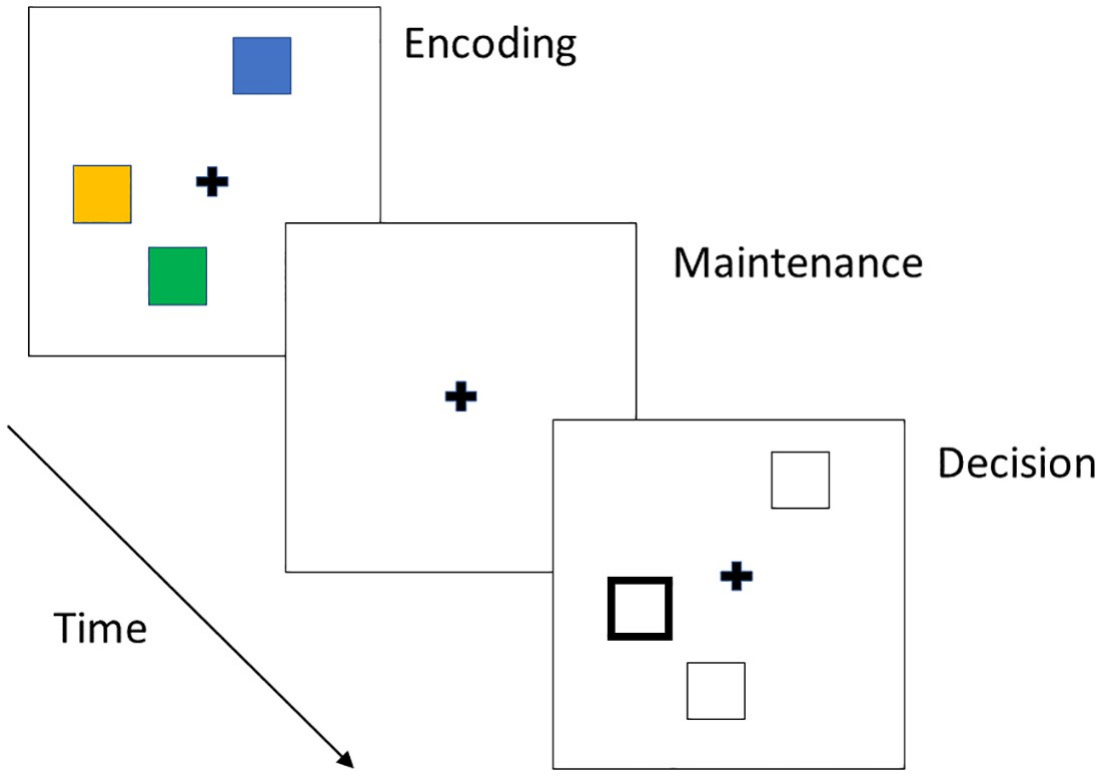
Continuous colour-report task. In a continuous colour-report task, participants are shown a stimulus array in an encoding phase, the array disappears during the maintenance period leaving for example only a fixation cross, and participants are cued with a probe in the decision phase. Here participants are probed with a location cue (solid outlined square) and asked to rotate a colour wheel to match the colour of the item at that location in the stimulus array. The timings are typically 0.1 to 2s for the encoding phase, 0.5 to 10s for the maintenance interval and 0.5 up to 2s for the decision phase. Other continuous report tasks vary in their combination of cue and recall attributes (e.g. location, colour, orientation).

In the continuous colour-report task illustrated in Fig 1, participants are shown a stimulus array in an encoding phase, the array disappears during a maintenance interval, and in a decision phase participants are cued with a location and asked to rotate a colour wheel to match the colour of the item at that location in the stimulus array. Similar experimental paradigms vary as to whether the cued or reported attributes are colours, orientations or locations [8, 19, 20]. The use of continuous reports, in which the reported variable is continuous rather discrete, has changed our understanding of the nature of working memory. These tasks have led to the proposal that, rather than working memory having a fixed capacity limit on the number of items that can be stored (e.g. between 3 and 7 items), the more fundamental limit is on the precision with which items can be recalled [6]. Precision has been found to decrease with load (number of items) [5]. Precision has also been found to decrease with length of the maintenance interval. For example, Rademaker et al. (2018) [21] tested memory for single grating orientation, patch color, and face identity items (generated via a continous circular latent space) across delays of 1, 3, 6 or 12s. Across all conditions, participants exhibited a clear decline in the precision of their working memories. The loss of precision over the maintenance interval is a finding that is consistent with gradual accrual of noise. Generally, the loss of precision has a stronger dependence on load than delay length and studies have found interactions such that the effect of delay length is magnified at higher loads [12, 19].

### 1.2 Models of working memory

Here we very briefly review other mathematical models of working memory. As this is a large research area we refer the reader to more substantial reviews [6, 11, 22] and here view the field as comprising either (i) “cognitive models” or (ii) “neuronal models”.

In the category of cognitive models Zhang and Luck [4] proposed a mixture model that accounts for two types of trial (i) guesses and (ii) responses based on the target item, where responses are modelled as circular Gaussians (Von-Mises densities) with an estimable precision parameter. This was then extended by Bays et al [5] to additionally account for (iii) responses based on non-target items. Such responses have become known as “swap errors” and this mixture model has also become known as the swap error model. It is implemented in the MixtureFit [5] and MEM toolboxes [23] and has been applied to data from a large number of empirical studies. It can provide estimates of swap error rates and guess rates, and can measure how precisions change with load and delay length. Further work in this vein includes the “variable precision” model which accommodates trial-to-trial variability in precision by modelling responses with a circular t-distribution rather than a circular Gaussian [24, 25]. These heavier-tailed distributions have called into question the need for guessing terms at all, as an apparent increase in guess rate with set size may simply reflect an increasing prevalence of low-precision representations. Such a “pure resource” model implies that there is no discrete item limit in working memory, rather that all items are encoded but with a variability in precision that depends on multiple factors including set size, alertness and covert shifts of attention [6]. More recently, Shurgin et al. [26] have proposed a Target Confusability Competition (TCC) model in which working memory performance can be accurately described by standard signal detection theory combined with an appropriately scaled and empirically determined psychophysical similarity function. TCC explains an impressive range of working memory phenomena and, as with the variable precision models, does not require guessing terms. It has also been proposed that the similarity function in TCC is closely related to the tuning functions of population coding models, but there is an ongoing debate as to the exact nature of this relationship [26, 27].

Foundational work with neuronal models describes, for example, how dopaminergic input to recurrent networks causes changes in excitatory and inhibitory channel conductances thereby switching circuits in prefrontal cortex into (and out of) dynamical regimes in which persistent cell activity can support working memory [28–30]. Generally, neuronal models are not usually directly fitted to empirical data but there are exceptions. One of these is the Dynamic Neural Field approach of Schoner and Spencer [18]. These models describe the activity of inhibitory and excitatory neurons arranged into one- or two-dimensional neural fields that also capture the dynamics of persistent cell firing. The interaction of multilple neural fields from different putative brain regions then explains a number of empirical phenomena including load effects, delay effects, load-by-delay interactions, and repulsion effects [31, 32]. An alternative modelling framework relies on a population coding perspective [33] in which stimulus information is encoded and decoded from the noisy activity of a large population of neurons each responsive to different stimulus features. Importantly, this explains the limited precision of WM as arising from a finite neuronal resource such as the number of neuronal spikes or neuronal energy budget. Other modelling work accommodates recent empirical findings that WM may not be instantiated by persistent cell firing but rather by more dynamic representations that wax and wane during maintenance intervals. For example, using spiking neural network simulations Fiebig et al. [34] show that a fast-expressing form of Hebbian plasticity can support WM using oscillatory burst activity, and Bouchacourt and Buschmann [35] show that a two-component architecture with sensory networks coupled to a central randomly connected recurrent network can support WM using mixed static and dynamic representations.

The Stochastic Attractor models (SAs) in this paper offer a level of description intermediate between cognitive and neuronal models. They are based on the mechanism of attractor dynamics but have no explicit neurons or synaptic dynamics. To a degree, this gives the models similar explanatory power but they are easier to fit to empirical data. As with cognitive models, the Stochastic Attractor approach provides a compact model of the recall probability density. In addition it provides an analytical expression (via the Fokker-Planck equation) for how this density evolves over time so, in this regard, provides a more parsimonious representation than mixture, TCC, variable precision and population coding models which require additional parameters at each delay length of interest. As we show in this paper, this also allows us to compute new and useful quantities such as the swap-error curve.

The focus of this paper is on modelling behavioural data from continuous-report visual working memory tasks and we provide Matlab code to support this (https://github.com/wpennyUEA/StochAttractVWM). However, in S1 Text (see also Discussion) we show how this framework could be generalised to a Multiple Attribute formalism that may reflect the distributed nature of neural processing during working memory tasks [36–39].

## 2 Methods

### 2.1 Attractor dynamics

This section reviews the Stochastic Attractor formalism proposed by Panichello et al. [12] as a model of working memory. The main idea behind stochastic attractors is that there are two distinct forces governing the dynamics of memory traces—the first being a stochastic force that causes traces to randomly diffuse over time, and the second being a deterministic force that directs traces towards attractor states (stable fixed points). Mathematically, a memory trace for attribute *x* (e.g. colour, location or orientation) evolves according to the Stochastic Differential Equation (SDE)

$$\begin{array}{c} dx=\beta g(x)dt+\sigma dw \end{array}$$ (1)

where *dw* is a stochastic diffusion term that follows a Wiener process [40, 41], *g*(*x*) is a deterministic flow function, with *σ* and *β* controlling the magnitudes of the two forces.

Eq 1 can be written as a dynamical equation governing the evolution of the probability density over *x*. This is known as the Fokker-Planck equation [42] and is given by

$$\begin{array}{c} \frac{\partial }{\partial t}p\left(x,t\right)=-\frac{\partial }{\partial x}\left[\beta g\left(x\right)p\left(x,t\right)\right]+\frac{\sigma^{2}}{2}\frac{\partial^{2}}{\partial x^{2}}p\left(x,t\right) \end{array}$$ (2)

If we discretise *x* into bins the above equation can be written as

$$\begin{array}{c} \frac{\partial }{\partial t}p\left(x,t\right)=Mp\left(x,t\right) \end{array}$$ (3)

where *p*(*x*, *t*) is the probability density (a [*B* × 1] vector), *M* is a [*B* × *B*] Markov transition matrix constructed from the flow and diffusion terms, and *B* is the number of bins. The results in this paper were obtained with *B* = 100. The density at time *t* is then given by

$$\begin{array}{c} p\left(x_{t}\right)=\text{exp}\left[Mt\right]p\left(x_{0}\right) \end{array}$$ (4)

where exp[] denotes the matrix exponential, and *p*(*x*_0_) is the initial density (after encoding). See Harrison et al. [43] and Deco et al. [44] for applications of the Fokker-Planck equation in systems neuroscience. More introductory material including applications of differential equation models in neuroscience and prototype models of working memory can be found in [45].

### 2.2 Flow functions

In their empirical data, Panichello et al. [12] found that memory reports clustered around specific values (similar findings have been made in other studies [14, 15]). This motivated them to employ a flow function with stable fixed points that could correspond to the cluster centres. These were identified by parameterising a flow function using a basis set and estimating the corresponding parameters from empirical data. The basis set comprised J first derivatives of the Von-Mises distribution each separated by 1 standard deviation

$$\begin{array}{c} g\left(x\right)=\sum_{j=1}^{J}w_{j}\phi^{\prime }(\frac{2\pi }{J}j,\frac{2\pi }{J}) \end{array}$$ (5)

where *ϕ* is the Von-Mises density parameterised by a mean and standard deviation and *ϕ*′ is its derivative. The parameters *w* are estimated when the model is fitted to participants responses. This produces a number of stable fixed points that correspond to the response biases. We refer to this as the Response-Bias flow function. In their empirical work, Panichello et al use *J* = 12.

**Fig 2:**
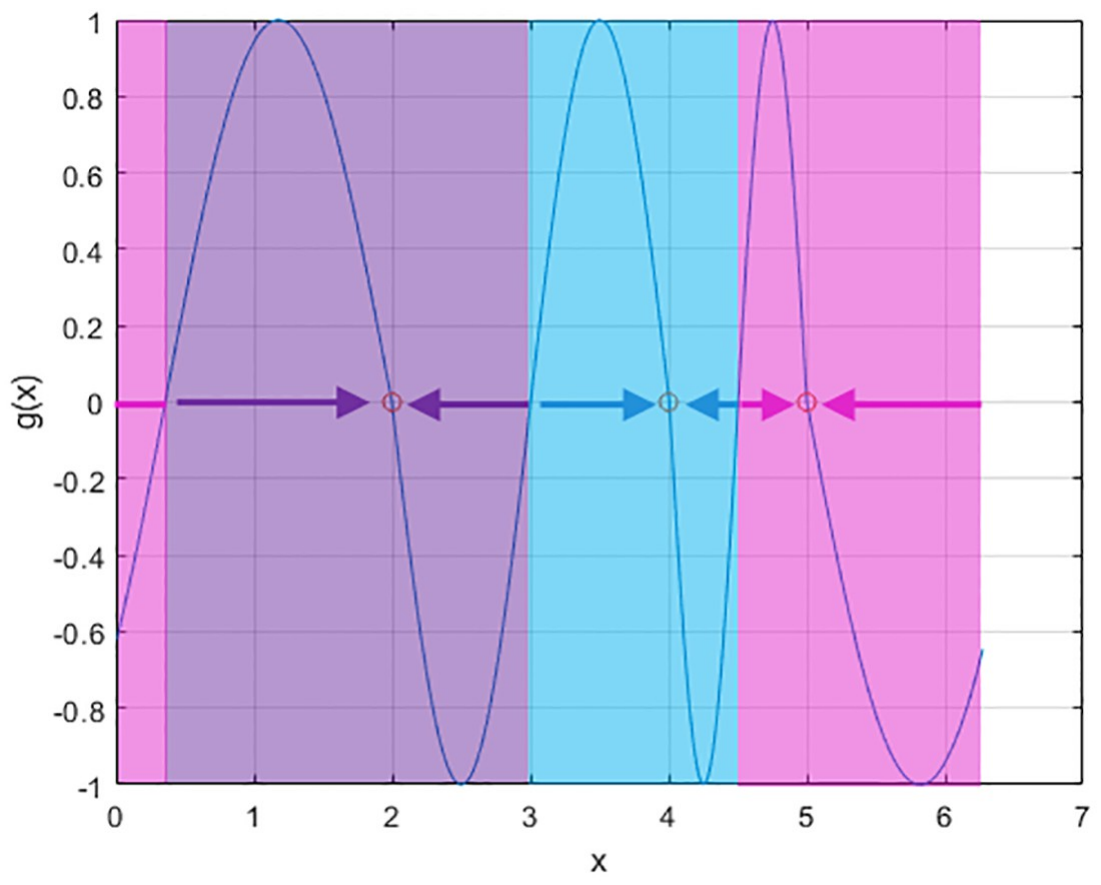
Flow function. This flow function (solid line) creates stable fixed points at x = 2, x = 4 and x = 5. If *x* is just above 2 it will experience a negative flow i.e. towards 2. Similarly if it is just below 2 it will experience a positive flow i.e. towards 2. Therefore *x* = 2 is a stable fixed point. Similarly for *x* = 4 and *x* = 5. More generally, stable fixed points occur at values of *x* for which *g*(*x*) is zero and the slope of *g*(*x*) is negative. The resulting basins of attraction and directions of flow are indicated by the coloured background and coloured arrows.

**Fig 3:**
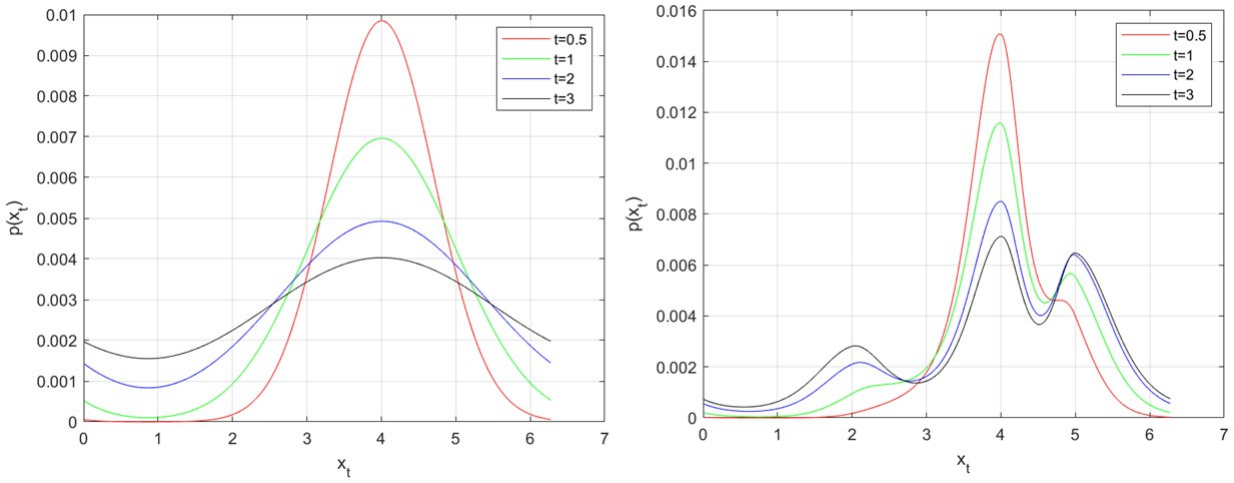
Evolution of memory traces. Memory traces evolve, from a delta function at initial state *x* = 4, according to the stochastic differential equation *dx* = *βg*(*x*)*dt*+ *σdw* with flow function *g*(*x*) from Fig 2, with diffusive noise parameter *σ* = 1, without the attractor (*β* = 0, left panel) and with the attractor (*β* = 1, right panel). Snapshots of the evolving probability distributions are shown at t = 0.5, 1, 2 and 3s. Without the attractor, memory of the initial state *x* = 4 is soon overwhelmed by diffusive noise. With the attractor, memory of the initial state is preserved via error-correcting attractor dynamics, with some later leakage of probability mass towards neighbouring stable fixed points (initially to *x* = 5 and later to *x* = 2).

In this paper we propose a different flow function. On each trial we choose the stable states to correspond to the values of the attributes to be remembered. For example, if we have 3 colours to remember with values *x* = 2, 4, 5 (e.g. from a colour wheel) then these stable states can be instantiated using the flow function shown in Fig 2. This is created by ensuring that the derivative of *g*(*x*) is negative at those values of *x*. We instantiate this by creating *g*(*x*) in a piecewise manner. The first piece corresponds to a negative sinewave, −sin(*x*), starting at the first value of *x* to remember with its frequency set so that a complete cycle will have been taken when it reaches the second value of *x*. This continues for all values of *x* until *g*(*x*) is complete. We refer to this as the Multi-Item flow function. This constructive approach is also advantageous from a statistical perspective as there are no parameters to estimate. The evolution of the probability density for this Multi-Item flow function is shown in Fig 3, with the attractor switched off (*β* = 0) in the left panel and switched on in the right panel (*β* = 1). Models with *β* = 0 are referred to as Pure Diffusion (PD) models. Flow functions of the sort shown in Fig 2 result in “error-correcting” dynamics because as memory traces drift away from stable fixed points they are pulled back by a flow force that increases in strength in proportion to the error (up to a limit). In this paper we will fit Response Bias, Multi-Item and Pure Diffusion models to empirical data and use a model comparison metric to assess which is best. Previously, Panichello at al. [12] have found evidence in favour of Response Bias rather than PD models.

**Fig 4:**
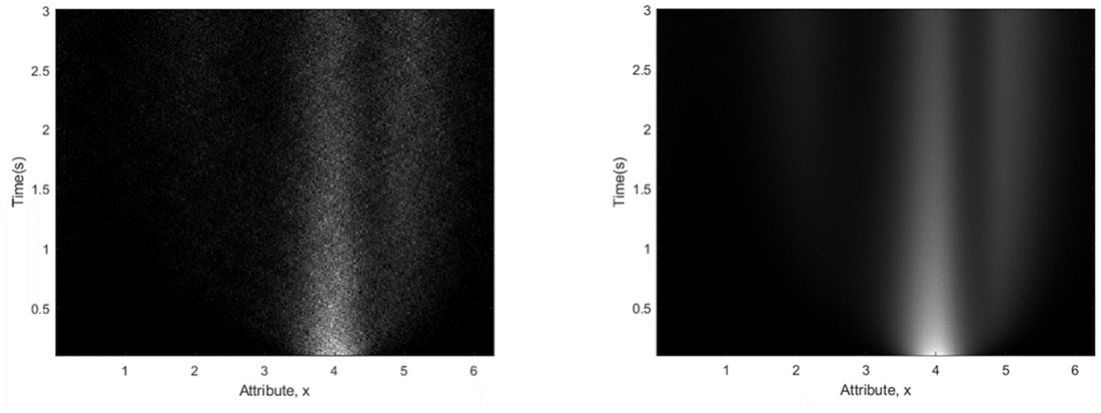
Solution of stochastic differential equation. Left Panel: 10,000 sample paths from the Euler Maruyama method, Right Panel: Fokker-Planck method. Snapshots of the Fokker-Planck density are show at selected time points in the right panel of Fig 3.

We close this section with reference to Fig 4 which compares solution of the stochastic differential equation (Eq 1) using the Fokker-Planck approach (right panel) with the Euler-Maruyama [41] stochastic integration method (left panel). The plots show good agreement, with the benefit of the Fokker-Planck being that we have a closed-form expression (Eq 4) for the density as a function of delay.

### 2.3 Encoding, maintenance and recall

Here we specify encoding densities as given by a delta function situated at the item to be remembered which is then convolved with a fixed-precision circular Gaussian

$$\begin{array}{c} p\left(x_{0}|z=j\right)=\text{exp}\left[\sigma_{e}D\right]\delta \left(x-c_{j}\right) \end{array}$$ (6)

where *D* is a [*B* × *B*] diffusion matrix, *c*_*j*_ is the *j*th item to be encoded, *δ*() is a delta function equalling unity when its argument is zero and zero otherwise, and *σ*_*e*_ quantifies encoding noise. This specification is a simplification of [12] which additionally included attractor dynamics during encoding. We then assume that memory traces evolve as a stochastic attractor with flow function *g*(*x*). This could correspond to that of a multi-item or response-bias model (see previous section). That is

$$\begin{array}{c} dx=\beta g(x)dt+\sigma dw \end{array}$$ (7)

The delay density after time *τ* is then

$$\begin{array}{c} p_{d}\left(x|z=j,\tau \right)=\text{exp}\left[M\tau \right]p\left(x_{0}|z=j\right) \end{array}$$ (8)

where matrix *M* summarises the action of the stochastic dynamics. To account for decision noise during recall, delay densities are convolved with a fixed-precision circular Gaussian

$$\begin{array}{c} p\left(x|z=j,\tau \right)=\text{exp}\left[\sigma_{r}D\right]p_{d}\left(x|z=j,\tau \right) \end{array}$$ (9)

where *σ*_*r*_ quantifies decision noise. In previous work [12], model comparisons showed that the inclusion of such a decision noise term was beneficial.

### 2.4 Guesses

Mixture model accounts of data from continuous report tasks [5] allow for the possibility that, on a proportion of trials, participants simply guess. This proportion can then be estimated using the mixtureFit.m algorithm [5] from the Analogue Report toolbox (www.bayslab.com) or similar code in the MemToolbox from www.visionlab.github.io/MemToolbox/ [23]. Here we adopt the same approach by incorporating an additive guessing term to the report likelihood. The likelihood of participant response *y*_*n*_ on trial *n* is then

$$\begin{array}{c} p\left(y_{n}|\theta \right)=\left(1-\lambda \right)p\left(x=y_{n}|z=\hat{i},\tau_{n}\right)+\lambda \frac{1}{2\pi } \end{array}$$ (10)

where *τ*_*n*_ is the maintenance interval on trial *n*, $\hat{i}$ indexes the target on trial *n*, and λ is the guessing (or “non-encoding”) probability, and *θ* are the model parameters—see below. We also consider models without guessing terms in which case

$$\begin{array}{c} p\left(y_{n}|\theta \right)=p\left(x=y_{n}|z=\hat{i},\tau_{n}\right) \end{array}$$ (11)

### 2.5 Swap errors

Similarly, mixture models of continuous report tasks also allow for “swap errors”, where participants report from the density of a non-target item. We could therefore use a likelihood that includes both guesses and swap errors

$$\begin{array}{c} p\left(y_{n}|\theta \right)=\left(1-\lambda -\alpha \right)p\left(x=y_{n}|z=\hat{i},\tau_{n}\right)+\alpha \frac{1}{m}\sum_{i=1}^{m}\;p\left(x=y_{n}|z=i,\tau_{n}\right)+\lambda \frac{1}{2\pi } \end{array}$$ (12)

where *i* indexes non-targets and *α* is the swap-error probability. This additional swap-error term does not need to be included for Multi-Item dynamics, as swaps are already accommodated by a process in which memory traces diffuse away from their initial state and are captured by another items’ attractor (see Fig 3 and next section). However, the Response-Bias model proposed in Panichello et al., and Pure-Diffusion models do include this additive swap error term.

### 2.6 Swap error curves

In the multi-item model (see right panel of Fig 3), a memory trace evolving from initial encoding at *x* = 4 is more likely to be captured by an attractor at *x* = 5 than an attractor at *x* = 2. That is, the probability density will tend to be higher at attractor states closer to the encoded item than at attractor states further away. Moreover, the probability mass captured by other attractors (the swap probability) tends to increase with time. This increase in swap errors with delay is also reflected in empirical data, for example, in plots of the *α* parameter in Fig 2C of Pertzov et al. [19]. Additionally, the density at non-target items can exhibit non-monotonic effects e.g. some of the probability mass captured at *x* = 5 later diffuses to *x* = 2. The multi-item model therefore seems well-suited to capturing swap-errors in empirical data without additional parameterisation.

Once the parameters of the multi-item model have been fitted to empirical data we can compute the probability mass lying in the basins of attraction of non-target items (e.g. pink and purple regions in Fig 2 if *x* = 4 is the target item). Moreover, because the stochastic attractor is a time series model (see Eq 4) swap error rates can be computed at arbitrary delay lengths (including those for which no empirical data was provided) without the need for additional parameterisation. This gives rise to *Swap Error Curves* which are generated by first fitting the multi-item model to empirical data, and then sampling responses as follows. On each simulated trial we (i) generate a stimulus array with cue and report attributes drawn from a circular uniform density (with minimum inter-item distance 30 degrees), (ii) compute the report density using Eq 9, and (iii) compute the probability mass in the basins of attraction of non-target items. This is repeated for *S* = 20 trials at each level of load and delay of interest. We then compute a mean swap error curve from the *S* simulations and confidence intervals from the standard error of the mean.

### 2.7 Model parameters

Model parameters govern encoding noise (*σ*_*e*_), attractor strength (*β*), diffusion strength (*σ*^2^), decision noise (*σ*_*r*_) and guess rates (λ). The Response-Bias and Pure-Diffusion models additionally have swap error rates (*α*). Response-Bias models additionally have a J-dimensional parameter vector *w* (with *J* usually set to 12 [12]) that weights basis functions for approximating the flow function (see Eq 5).

In order to constrain parameter estimates within a sensible range our optimisation algorithm works with “latent” parameters, *θ*, which define parameters through nonlinear transforms as follows. To produce the parameters *β*, *σ*, *σ*_*e*_, *σ*_*r*_ we use scaled sigmoidal transforms of latent parameters so that the parameters of interest take on a range of values similar to that found in previous empirical work. These are [0, 15] for *β*, [0.01, 1.5] for *σ*, and [0.01, 0.1] for *σ*_*e*_, *σ*_*r*_. We use a softmax transform for *w* (so that the sum over weights equals 1). The use of latent parameters and sigmoidal/softmax transforms are widely used in other behavioural and neuronal modelling contexts [46–48].

In this paper we allow the diffusion parameter *σ* to vary across load. This is in line with the view that internal representations of sensory stimuli are noisy, and that the level of this noise increases with the number of stimuli in memory. This dependence on set size is also shared by models of attention [6]. It is, of course, possible to allow additional parameters to vary with load as in [12] and we return to this issue in the discussion.

Guess rates are also allowed to vary across load *x*_*l*_, delay *y*_*d*_ and their interaction using a linear model (Eq 13) where *x*_*l*_ and *y*_*d*_ are normalised to have zero mean. This was again implemented using the latent parameter approach. For guess rates we have

$$\begin{array}{ccc} a_{ld} & = & \rho_{1}+\rho_{2}x_{l}+\rho_{3}y_{d}+\rho_{4}x_{l}y_{d}\\ \lambda_{ld} & = & \lambda_{max}Sigmoid\left(a_{ld}\right) \end{array}$$ (13)

for load level *l*, delay level *d* with λ_*max*_ = 0.2. Inferences about main effects and interactions over a group of subjects can then be implemented using t-tests across estimates of *ρ*_2_, *ρ*_3_, *ρ*_4_. A similar parameterisation is used for swap rates for the Response-Bias and Pure-Diffusion models.

### 2.8 Prior, likelihood and joint likelihood

In what follows N(m,R) is a Gaussian distribution over random variable *x* with mean vector *m* and precision matrix *R*. We define a Gaussian prior over the latent parameters

$$\begin{array}{ccc} p(\theta ) & = & N(w_{0},R_{0})\\ R_{0} & = & diag(r_{0})\\ r_{0} & = & {\left(\frac{1}{\sigma_{0}}\right)}^{2} \end{array}$$ (14)

where the prior mean *w*_0_ is a zero vector, *R*_0_ is a diagonal prior precision matrix with prior standard deviation set to *σ*_0_ = 1.68. We have *P* model parameters. When *θ* are sigmoidally transformed this gives an approximately uniform distribution over the parameters of interest.

Empirically, we are given response data *Y* = {*y*_1_, .., *y*_*n*_, …, *y*_*N*_} over *N* trials where the data on trial *n* is the participants ‘response’ *y*_*n*_, that is, their estimate of the target orientation, location or colour (depending on the experimental paradigm). The log-likelihood is therefore

$$\begin{array}{ccc} L(\theta ) & = & \sum_{n=1}^{N}\;L_{n}\\ L_{n} & = & \text{log}\;p(y_{n}|\theta ) \end{array}$$ (15)

where the definition of *p*(*y*_*n*_|*θ*) depends on whether we include guessing terms (Eq 10 for guesses, Eq 11 for no guesses) or both guessing and swap error terms (Eq 12). We emphasise that the multi-item model does not require an additive swap term as swaps naturally occur in the model (see above). The log of the joint likelihood and prior, to be used in model fitting, is then

$$\begin{array}{ccc} J(\theta ) & \equiv & \text{log}[p(Y|\theta )p(\theta )]\\ & = & L(\theta )+\text{log}\;p(\theta ) \end{array}$$ (16)

The computational bottleneck in model fitting is determined by computation of the likelihood which is, in turn, dominated by computation of the matrix exponential in Eq 4. For the RB and PD models we can compute *A*_*ld*_ = exp(*M*_*l*_*t*_*d*_) at load *l* and delay length *d*, a quantity which is constant for all trials at those loads and delays. This quantity can therefore be pre-computed prior to optimisation thus reducing model-fitting time. But for the MI model this is not the case because the flow function is trial-specific, which means that *M* and therefore *A* is trial-specific. This means that the MI model takes longer to fit and model fitting time is proportional to the number of trials.

### 2.9 Model fitting

Model parameters were estimated by maximising *J*(*θ*) using a Quasi-Newton algorithm implemented in the function fminunc.m from Matlab’s optimisation toolbox. Unless otherwise stated below, the optimisation was run from a single initialisation (a random sample from the prior) for a maximum of 256 iterations. Preliminary experiments on MI and PD models showed only very weak dependence on parameter initialisation (not enough to influence the results of model comparison)—but see below for comments on RB models.

Model fitting produces estimated parameter values $w_{k}\equiv \hat{\theta }$ for subject k. At the end of optimisation we compute a full posterior precision matrix using the outer-product approximation

$$\begin{array}{c} R_{k}=R_{0}+\sum_{n=1}^{N}(\frac{dL_{n}}{d\theta }){\left(\frac{dL_{n}}{d\theta }\right)}^{T} \end{array}$$ (17)

The posterior covariance is then computed as $C_{k}=R_{k}^{-1}$. As an aside we note that if one is merely interested in computing posterior variances, rather than covariances, we need to take the diagonal *after* the matrix inverse (diagonalising before inverting does not give the correct result). Overall, within-subject model fitting for subject *k* produces the approximate posterior

$$\begin{array}{ccc} q(\theta_{k}) & \equiv & p(\theta_{k}|Y_{k})\\ & = & N(w_{k},R_{k}) \end{array}$$ (18)

### 2.10 Model comparison

In the empirical work in this paper, we compare (A) Multi-Item models, where the stable fixed points correspond to the multiple items to be remembered (as proposed in this paper), to (B) Response-Bias models where the stable fixed points correspond to response biases (as proposed in [12]), to (C) purely diffusive models which have no deterministic force directing memory traces to stable fixed points (*β* = 0).

For within-subject model fitting, the Bayesian log evidence for model *m* fitted to data from subject *k* is given by the Laplace approximation [13]

$$\begin{array}{ccc} F_{k}\left(m\right) & \equiv & \text{log}\;p(Y_{k}|m)\\ & \approx & J\left(w_{k}\right)+\frac{1}{2}\text{log}\left|C_{k}\right|+\frac{P}{2}\text{log}\left(2\pi \right) \end{array}$$ (19)

where $w_{k}=\hat{\theta }$ are the estimated values from model fitting, *C*_*k*_ is the posterior covariance matrix, |*C*_*k*_| its determinant, *P* is the number of model parameters, and *Y*_*k*_ is the response data from subject *k*. Given the model evidence we can then use Bayes rule to compute the posterior distribution over models

$$\begin{array}{c} p\left(m|Y_{k}\right)=\frac{p\left(Y_{k}|m\right)p\left(m\right)}{\sum_{m^{\prime }}p\left(Y_{k}|m^{\prime }\right)p\left(m^{\prime }\right)} \end{array}$$ (20)

assuming a uniform prior *p*(*m*). When comparing two models we can use Bayes factors, *BF*_*ij*_ = *p*(*Y*_*k*_|*m* = *i*)/*p*(*Y*_*k*_|*m* = *j*) [49]. Group Log Bayes Factors, *G*_*ij*_ can then be computed as follows

$$\begin{array}{c} G_{ij}=\sum_{k=1}^{K}\left[F_{k}\left(i\right)-F_{k}\left(j\right)\right] \end{array}$$ (21)

### 2.11 Cluster model

We also analyse data from the Colour-Report task [12] to identify clustering of reports around response biases (see “Colour-Report Task” in the results section below for the reason why). We used a matlab implementation (https://github.com/chrschy/mvmdist) of a Mixture of Von-Mises model [50] which fits the colour report data using an Expectation-Maximisation algorithm [50]. For each putative number of clusters, *c*, we computed the Bayesian Information Criterion

$$\begin{array}{c} BIC\left(c\right)=L-\frac{P}{2}\text{log}\;N \end{array}$$ (22)

where *L* is the log-likelihood of the cluster model, *N* is the number of data points and *P* is the number of model parameters (as each cluster has a mean, precision and frequency parameter we have *P* = 3*c*).

### 2.12 Empirical data

This paper analyses previously acquired data from two continuous report visual working memory experiments where reports are given at multiple loads and delay lengths. The generic structure of these tasks is shown in Fig 1.

#### 2.12.1 Location-report task

Schneegans and Bays 2018 [8] present results from ten participants performing a location report visual working memory task. After the presentation of a fixation cross, participants were presented with an array of 1, 2 or 4 coloured disks (chosen from the colours red, blue, yellow and green). The array was presented for 2s (encoding period) and disks were located on an invisible circle with a fixed radius from the fixation cross. The minimum distance between neighbouring items was 30 degrees. This encoding period was followed by a pattern mask display (visible for 0.1s) and a delay period when just the fixation cross was present. The total memory delay including mask display was 0.5, 1, 2 or 4s. The fixation cross was then replaced with a centrally presented response cue in the form of a colored disk, which matched the color of one of the disks from the encoding array. Participants then had to make a saccadic eye movement to the memorised location of the disk. The 4 delay lengths crossed with 3 load levels gives rise to 12 experimental conditions. There were approximately 19 trials per condition leading to a total of 230 or so trials per subject. Full details of the paradigm are available in [8] and the data we refer to are from “Experiment 1” of that paper. These data are available from https://www.paulbays.com/publications.php.

#### 2.12.2 Colour-report task

This data come from an experiment in which 90 subjects performed a color-report visual working memory task [12] in which they reported the colour of a spatially cued sample after a variable delay (1s or 7s). Each sample was located at one of eight possible spatial locations. The colours were drawn from a uniform distribution on a colour circle with the caveat that colours presented on the same trial had to be at least 22 degrees apart in colour space. Reports were made by adjusting the hue of the response probe by rotating a response wheel using a mouse (see Fig 1 in [12]). The mapping between wheel angle and colour was rotated on each trial to prevent spatial encoding of colour memories. Additionally, there were two load levels comprising either 1 or 3 items. Data for each subject comprises up to 200 trials in all, with 50 in each of four conditions. These conditions vary over the combinations of 2 load levels (1 and 3 items) and 2 delay lengths (1s and 7s). Overall, after rejecting subjects who made too many guesses, data was available for 30 subjects who did the task in a laboratory setting and 60 who did it online. For further details, see the description of Experiment 1a in [12].

## 3 Results

In what follows we first fit multi item, response bias and pure diffusion models to synthetic data in order to validate our parameter estimation and model comparison procedures and to assess their efficacy as a function of sample size (number of data points). We then fit these models to empirical data from colour and location report tasks. Finally, we compare models with and without guess rates, as this has become an important distinction in the WM literature.

### 3.1 Synthetic data

We generated synthetic data from Multi-Item (MI), Response-Bias (RB) and Pure-Diffusion (PD) models. Data was generated for ten synthetic subjects with 250 trials per condition where the conditions varied over the combinations of load (1 or 3 items) and delay (1 or 7s) as in [12]. This “Large Dataset” therefore comprised 1000 trials per subject—see below for “Small Dataset”. True model parameters were *β* = 7 (zero for the pure-diffusion model), *σ*^2^ = 0.25 for load 1 and *σ*^2^ = 1 for load 3, *σ*_*e*_ = 0.05 and *σ*_*r*_ = 0.05. For the Response-Bias model, the true stable fixed points were set to 1.1, 2.9, 4.3 and 5.9 (similar to the cluster peaks in Fig 1C in [12]) and *J* = 12 basis functions were used to approximate the flow functions (see Eq 5).

For each simulated trial, location and colour cues were drawn from a uniform circular distribution, but with a minimum inter-item separation of 30 degrees. In order to focus on the identification of attractor parameters we set the true guess rate to zero (at all levels of load and delay) and the true swap rates to zero for the RB and PD models (for all levels of load and delay). We fitted the MI (*P* = 9 parameters), RB (*P* = 25) and PD (*P* = 12) models to each simulated data set.

**Fig 5:**
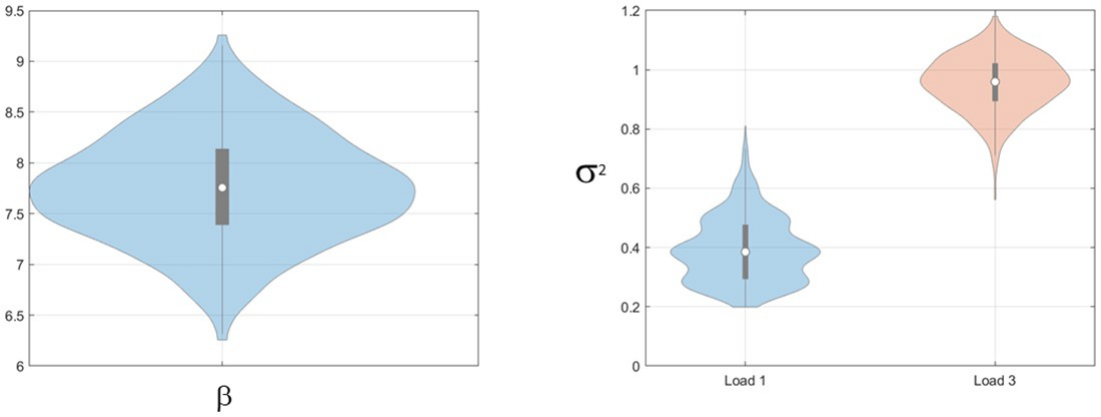
Synthetic data: Estimates of attractor parameters from multi-item model. Left Panel: Bootstrapped distributions of mean attractor strength, *β*,. True value was 7. Right Panel: Bootstrapped distributions of mean diffusion parameters, *σ*^2^, at loads 1 and 3. True values were 0.25 and 1.

**Fig 6:**
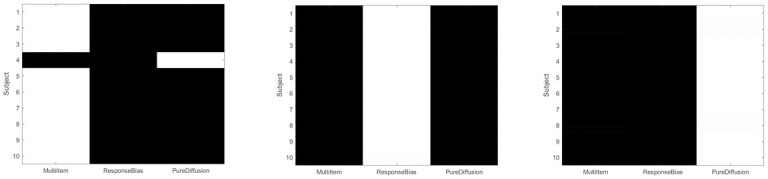
Synthetic data: Model comparison. The gray scales indicate the (posterior) probability (see Eq 20) that the data were generated from Multi-Item, Response-Bias or Pure-Diffusion models, with 1 shown in white and 0 in black (rows in each image sum to 1). The left, middle and right panels indicate simulations for which the true model was Multi-Item, Response-Bias, and Pure-Diffusion, respectively. Overall, we see that the correct model is identified in 29/30 simulations (see main text).

Model parameters and model evidence were then estimated as described in the methods section. Bootstrapped estimates of the mean estimated parameter values for the Multi-Item model are plotted in Fig 5 and show reasonable agreement with the true values. Fig 6 shows the results of the model comparison. Overall, we see that the correct model is identified in 29/30 simulations. For the simulation in which the true model did not have the highest model evidence, data from a multi-item model was attributed to a pure-diffusion model (left panel in Fig 6).

In more detail, Table 1 below shows the group log Bayes factors of the fitted model with respect to the best fitted model. The entry -45 (which is on average -4.5 per subject), for example, means that the MI model of the PD data is on average exp(−4.5) = 0.011 times as likely as (or about 90 times less likely than) the PD model of the PD data.

**Table 1:** Group log bayes factors for large synthetic dataset and within-subject model fitting. The entries in the table are group log Bayes Factors, *G*_*ij*_ (see Eq 21), of fitted models with respect to the best fitted model (a 0 indicates the best fitted model). The true models are correctly identified.

True Model	Fitted Model		
	MI	RB	PD
MI	0	-1948.5	-1066.1
RB	-3620.1	0	-3634.9
PD	-45.0	-1002.6	0

Informally, we note that model identification is possible for the following reasons. Data from the Multi-Item (MI) and Response-Bias (RB) models are not easily confused because the stable fixed points are trial-specific for MI but not for RB. Therefore no RB parameter setting can account for MI data, and MI parameter settings can’t account for RB data (as the fixed points will be in the wrong place—they can’t move from trial-specific values). Similar arguments apply to the Pure-Diffusion (PD) and RB models.

So far, so good. However, empirical datasets typically contain far fewer than 250 trials per condition. We therefore repeated the above simulations but with a “Small Dataset” with 50 trials per condition per subject. Whilst the model fitting and inference procedures worked well for the MI and PD models they were only able to correctly identify the RB models for 5 out of 10 subjects. Additionally, estimates of the RB flow-function were compromised. Reducing the number, *J*, of basis functions parameterising the RB flow function (see Eq 5) did not help.

This therefore motivated us to run a single optimisation using data from all 10 subjects, resulting in 500 trials per condition. These “Group-Fitted” results are presented in Table 2 and, unsurprisingly, provide excellent model comparison results.

**Table 2:** “Group-Fitted” log bayes factors for small synthetic dataset. he entries in the table are log Bayes Factors with respect to the best fitted model (a 0 indicates the best fitted model). The true models are correctly identified.

True Model	Fitted Model		
	MI	RB	PD
MI	0	-269.2	-153.8
RB	-1028.7	0	-1023.8
PD	-4.6	-122.3	0

We then ran model fits for individual subjects data using a parameter initialisation from the group-fitted solution. This worked well for the MI and PD models but only 6/10 RB models were correctly identified. This shows that careful model initialisation, to help avoid local maxima, is not always sufficient for correct model identification. Indeed, the problem here is not one of local maxima as the log-likelihoods of the RB models were maximal in all 10/10 subjects. Rather, the signal to noise ratio is not high enough in single subject data to correctly identify RB models at the within-subject level. Overall, the findings from this section show that MI and PD models can be correctly identified at the within-subject level, but that RB models can only be reliably identified at the group level.

### 3.2 Colour-report task

Here we use the data from Panichello et al. [12]. We fitted three models to each subject’s data (i) the Multi-Item model with guessing term, (ii) the Response Bias model with guessing and swap error terms and (iii) the Pure Diffusion model with guessing and swap error terms. For each model the diffusion noise level was also allowed to vary across load.

We first report results from a “group-fitted” model in which models are fitted to data from all subjects. As local maxima are of greatest concern for the RB model we ran 8 optimisations, each with a different initialisation, and selected the model with the highest joint likelihood. The MI and PD models were run from a single initialisation. The RB model was identified as the best with a Log Bayes Factor of 556.6 wrt to PD and 600.4 wrt MI. These results are in agreement with [12] in that RB has greater model evidence than PD.

**Fig 7:**
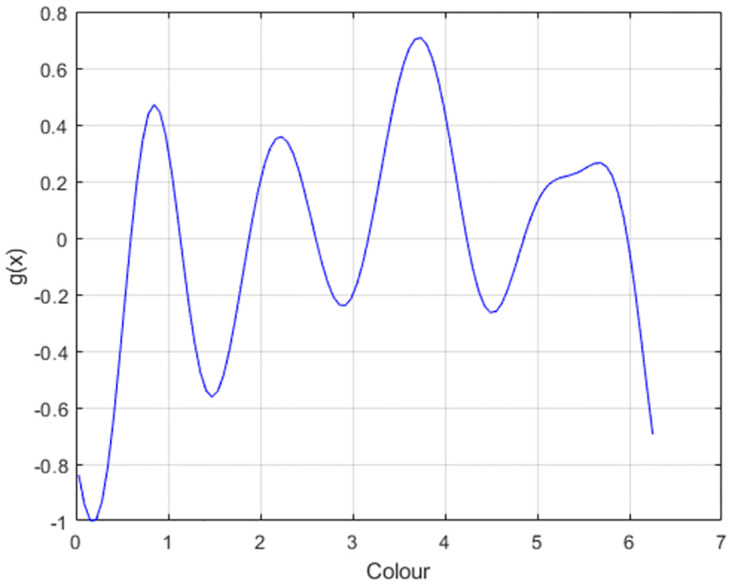
Colour report task: Estimated flow function for group-fitted response-bias model. This flow function was parameterised according to Eq 5. The zero crossings correspond to stable fixed points and are in similar positions to cluster peaks in response histograms, consistent with the idea that clustering of responses is driven by attractor dynamics.

Fig 7 shows the estimated flow function. This has stable fixed points (zero-crossings) close to the cluster peaks in Fig 1C in [12], consistent with the idea that attractor dynamics creates the clustering of participant responses. To assess potential between-subject variability we then fitted models to data from individual subjects, but initialising model parameters to the group-fitted solution to help avoid local maxima (as in the previous section on simulated data). Despite this, model comparison resulted in the MI model being favoured in 65/90 subjects and PD in 25/90 subjects. The average log Bayes factors in favour of MI were 5.5 wrt PD and 72.1 wrt MI.

**Fig 8:**
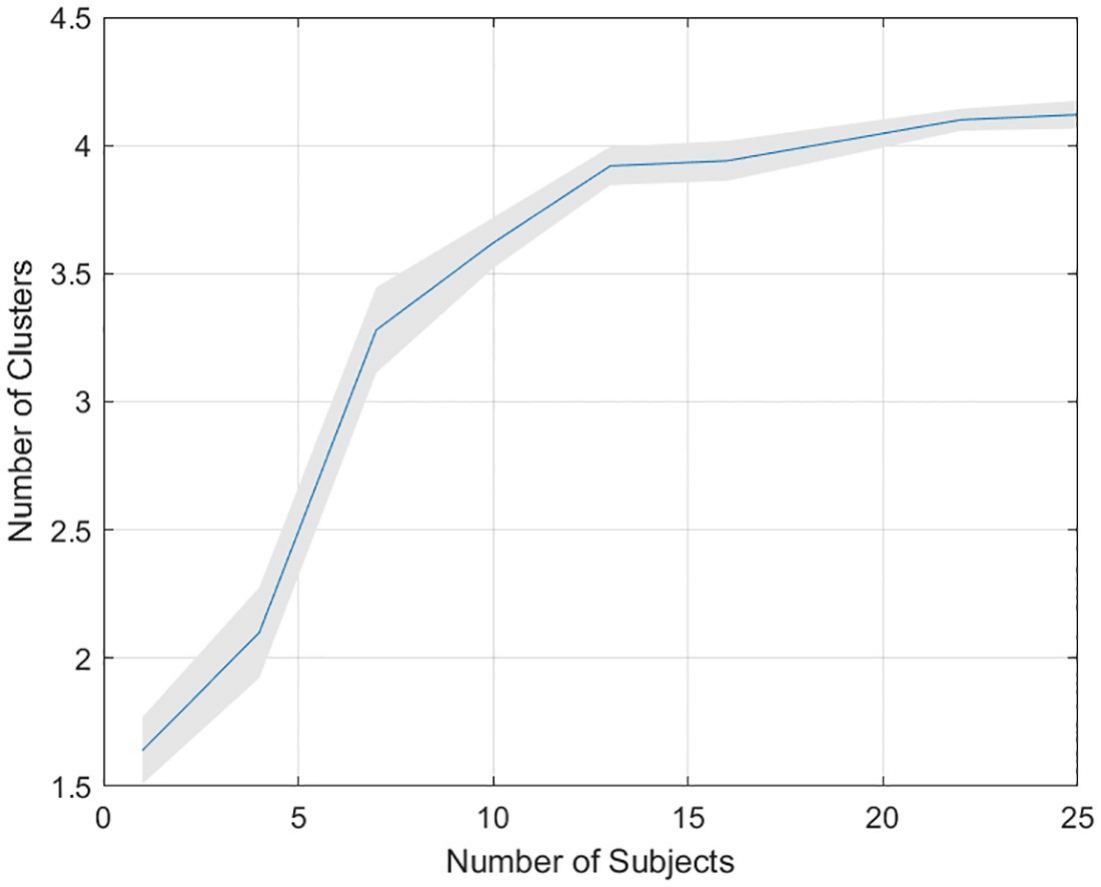
Colour report task: Clustering of responses in group data. The figure plots the mean number of inferred clusters versus the number of subjects’ data used. Inference was made using the Bayesian Information Criterion with a Mixture of Von-Mises clustering model (see section 3.11.

To find out what is driving the discrepancy between group-level and subject-level findings, we implemented a cluster analysis on the 90-subject empirical colour report data using a Mixture of Von-Mises model [50] (described in the methods section). This was repeated using data from a number of subjects that varied from 1 to 25 and the Bayesian Information Criterion (BIC) was used to compute the optimal moder order. At each group size we ran 50 replications where, at each replication, data was drawn from subjects chosen randomly without replacement from the 90 subjects available. The results in Fig 8 show that clustering of participant’s responses around biases is not evident given data from single subjects. Indeed, the emergence of the 4 clusters that are clearly evident in Fig 1c of [12] (computed using data from 90 subjects) does not appear until data from about 10 subjects are used. Overall, these results show that response biases are clearly manifest at the group level. But at the subject level, the attraction of memory traces toward target and non-target items, as captured by the Multi-Item model, is a stronger effect. These findings are consistent with the individual data being underpowered to detect response biases, and consistent with our findings from synthetic data (see above section).

**Fig 9:**
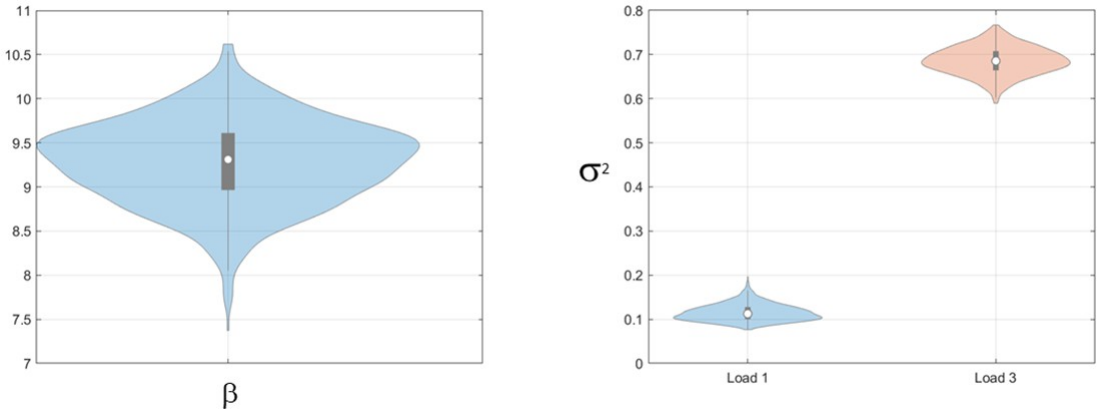
Colour report task: Attractor parameters for multi-item model. Left panel: Bootstrap distribution of mean attractor strength *β*, Right panel: Bootstrap distributions of mean diffusion parameters, *σ*^2^, at loads 1 and 3.

**Fig 10:**
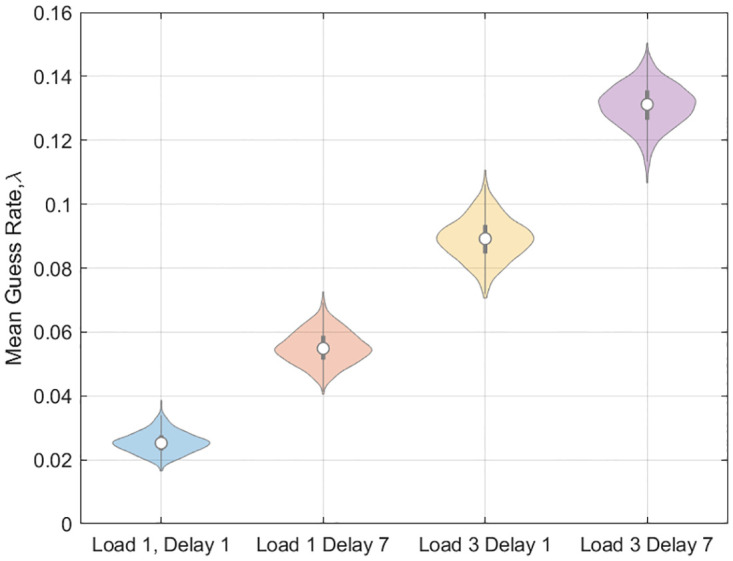
Colour report task: Guess rates for multi-item model. Bootstrapped distributions of guess rate at two levels of load and delay.

**Fig 11:**
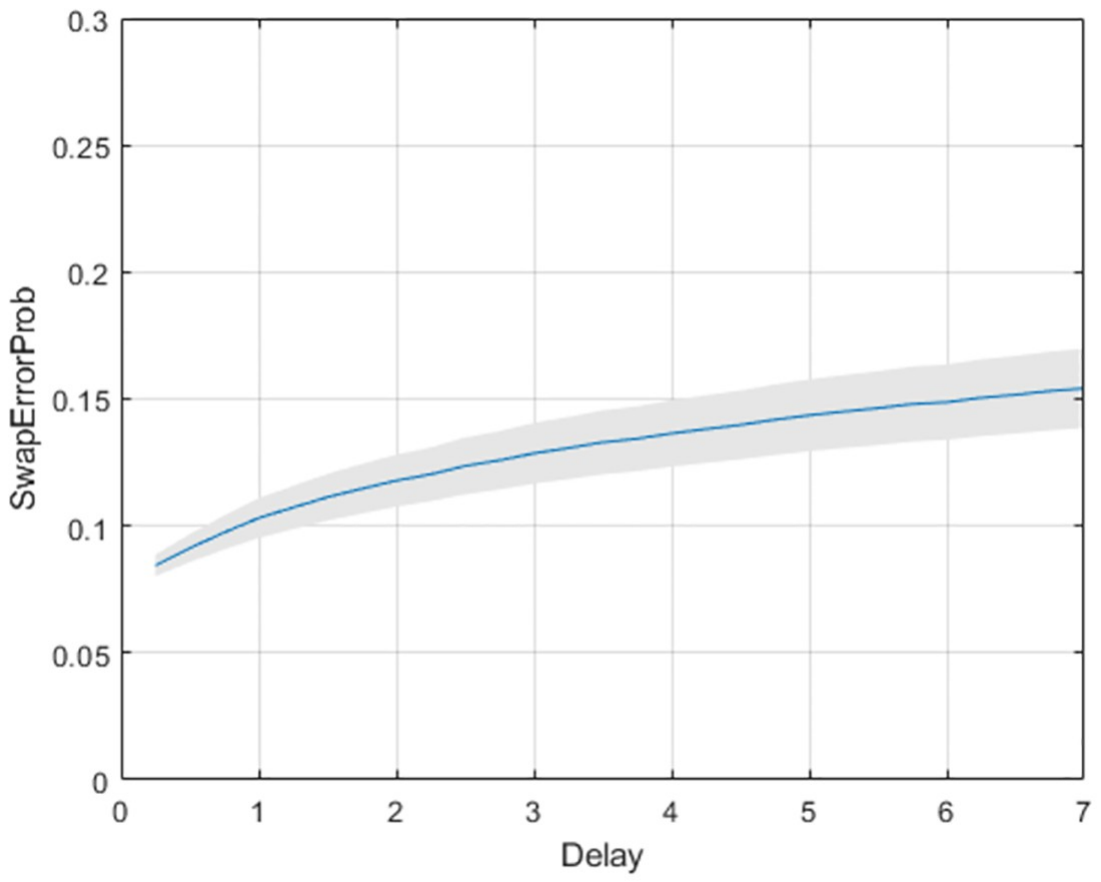
Colour report task: Swap error curves from multi-item model. Mean swap error curve over group of subjects for memorising 3 items. The grey shading indicates the confidence interval.

Figs 9–11 show bootstrapped estimates of the mean attractor strength, diffusion strength, guess rates and swap curves for the within-subject Multi-Item models. Diffusion strength increases with load (paired t-test on log parameter estimates, *t*(89) = 17.0, *p* < 10^−6^). The ratio of the strength of the flow force, *β*, to the diffusion force, *σ*^2^, was estimated to be 134 at load 1 and 21.2 at load 3, indicating that the relative strength of the attractor is reduced at high load. We return to this issue in the discussion.

The guess rates are more or less identical to those reported in [12] (Supplementary Fig 10). We found a main effect of load (*t*(89) = 14.2, *p* < 0.001), a main effect of delay (*t*(89) = 6.91, *p* = < 0.001) but no interaction (*t*(89) = 0.78, *p* = 0.439). The swap rates are higher than those reported in [12], possibly due to the different way in which swaps are defined (see methods section).

### 3.3 Location-report task

Here we use the data from Schneegans and Bays’ location-report task [8]. We fitted three models to each subject’s data (i) the Multi-Item model with guessing term, (ii) the Response Bias model with guessing and swap error terms and (iii) the Pure Diffusion model with guessing and swap error terms.

We fitted an RB model to data from all 10 subjects and ran the optimisation from 8 different initialisations. The MI and PD models were also fitted to this group data. The preferred model was MI with a log Bayes factor of 130.8 wrt RB and 113.9 wrt PD. We then used this group-fitted solution to initialise model fits to individual subject data. We found that MI was best for 10/10 subjects with a mean log Bayes factor of 80.8 wrt RB and 38.8 wrt PD.

There is therefore no evidence for attractor dynamics toward response biases within this data set. These results do however support the hypothesis of attractor dynamics toward multiple items. Moreover, this is favoured over the alternative hypothesis of a pure diffusion process. These results therefore agree with Panichello et al. in that working memory maintenance is not a purely diffusive process. Rather, error-correction is embodied within attractor dynamics that contain both diffusive and attractive components.

**Fig 12:**
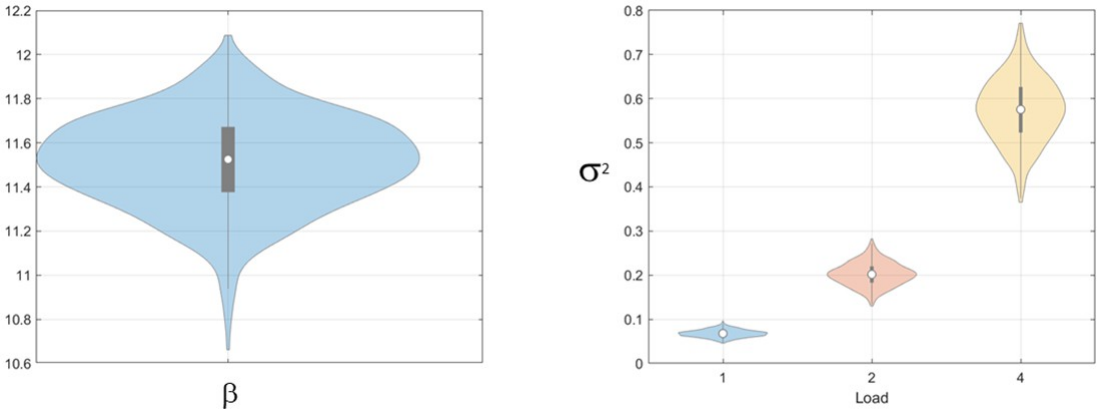
Location report task: Attractor parameters for multi-item model. Left panel: Bootstrap distribution of mean attractor strength *β*, Right panel: Bootstrap distributions of mean diffusion parameters, *σ*^2^, at loads 1, 2 and 4.

The diffusion parameter was allowed to vary with load and we indeed observed a main effect of load (using an F-test on log-transformed parameter estimates, *F*(2, 29) = 57.4, *p* < 0.001). Fig 12 show estimates of the attractor and diffusion strengths which are similar to those shown in Fig 6 of Panichello et al. [12]. Diffusion strength increases with load leading to the weakening of relative attractor strength with load (*β*/*σ*^2^ = 197 at load 1 and reduces to 68.1 at load 2 and 24.6 at load 4).

**Fig 13:**
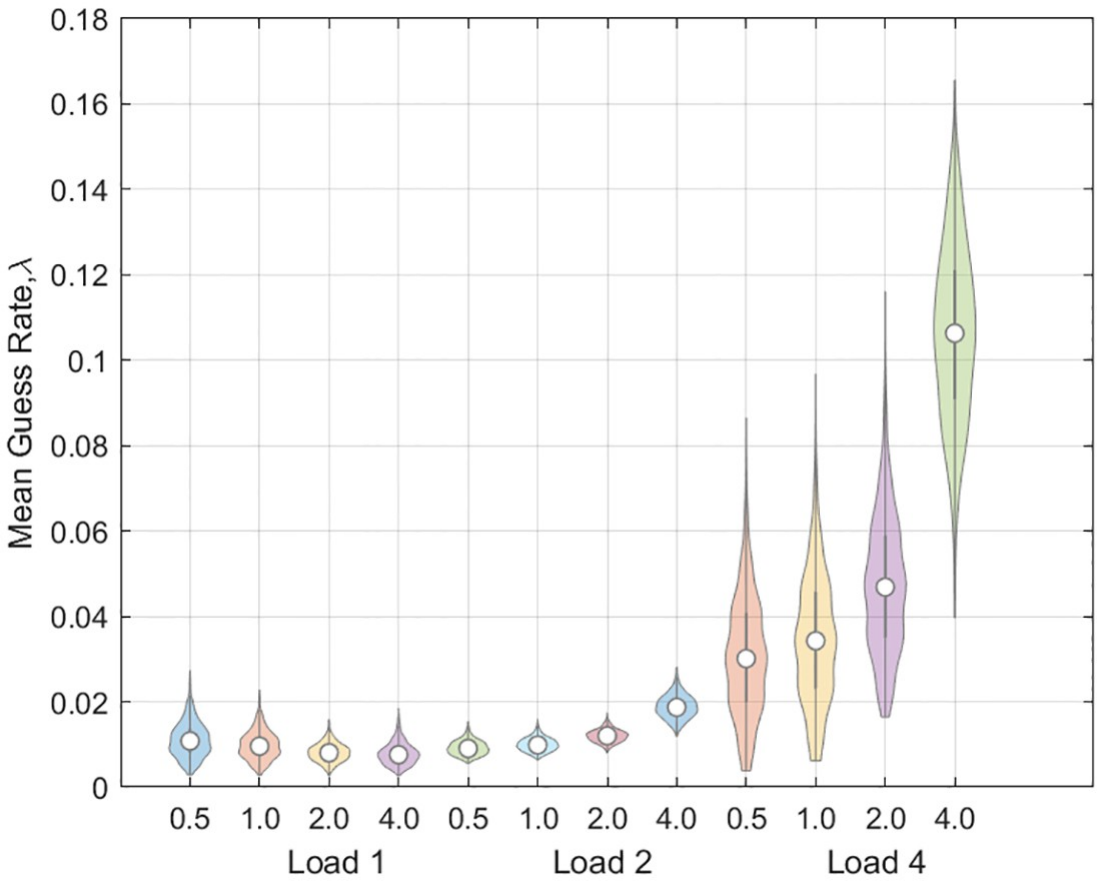
Location Report Task: Guess Rates from Multi-Item model. Bootstrapped distributions of mean guess rate at various combinations of load and delay (0.5, 1, 2 or 4 seconds).

**Fig 14:**
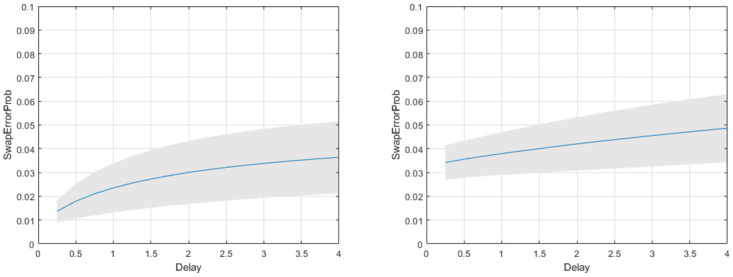
Location report task: Swap error curves from multi-item model. Mean swap error curve over group of subjects for memorising 2 items (left panel) and 4 items (right panel). The grey shading indicates the confidence interval.

For the guess rate, see Fig 13, we found a main effect of load (*t*(9) = 2.70, *p* = 0.024), a main effect of delay (*t*(9) = 4.32, *p* = 0.002) and an interaction (*t*(9) = 2.74, *p* = 0.023). Fig 14 shows the swap error curves at loads 2 and 4 as estimated by the Multi-Item model (there are, of course, no swaps at load 1).

Schneegans et al. [8] fitted the swap error mixture model to this data and the overall swap error rate was reported to be 2.2%. The swap error curves from the Multi-Item model estimate swap errors to be between 1 and 5% depending on load and delay (see Fig 14) which are broadly consistent.

### 3.4 Models without guesses

In this section we again fit the multi-item stochastic attractor model to empirical data but this time allowing for model variants without guessing terms. Specifically we compare three types of model; (i) a model MG0 without guesses, (ii) a model MG1 with a single extra parameter governing average guess rate (over load and delay), and (iii) a model MG4 with four extra guess rate parameters that allow guess rate to vary across load and delay.

**Fig 15:**
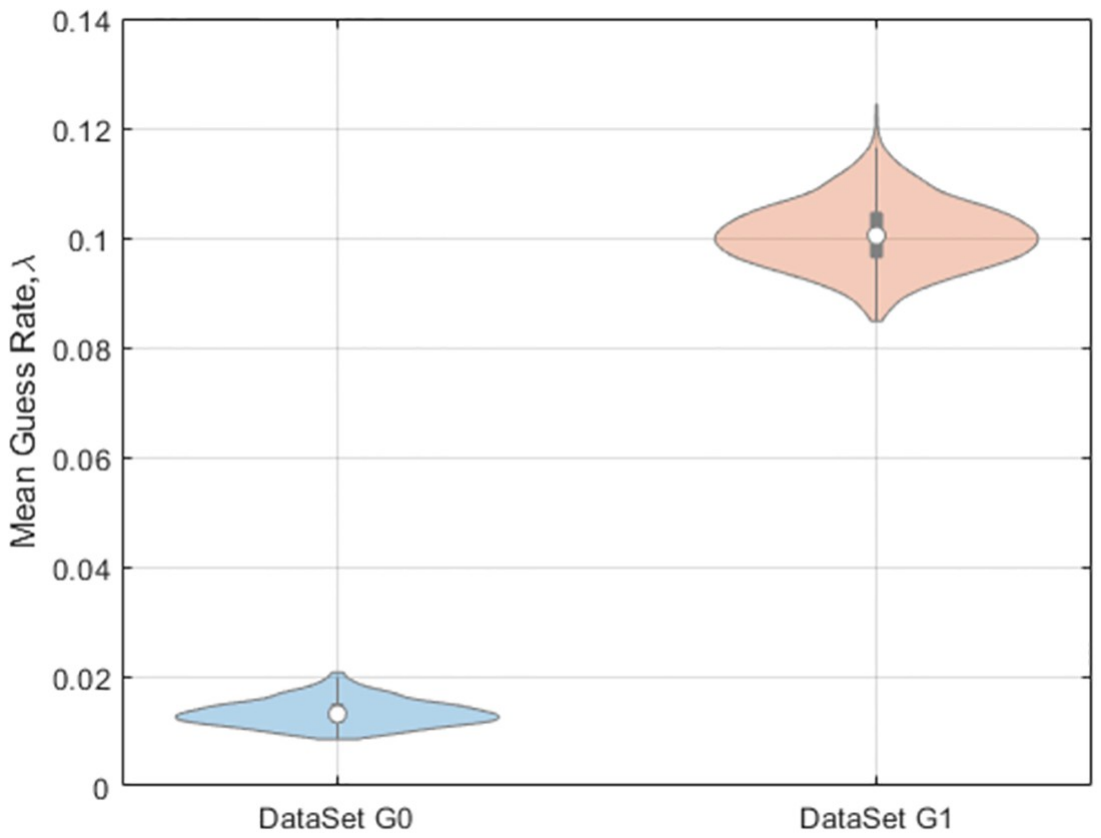
Estimated guess rates on synthetic data. for data set G0 (true guess rate = 0) and data set G1 (true guess rate = 0.1). Guess rates were estimated using a multi-item stochastic attractor model with a single guess rate parameter (referred to as model MG1). See main text for further details.

Before analysing the empirical data we first fit the models to simulated data where the ground truth is known. We consider simulated data with true guess rates of λ = 0 (data set G0) and (ii) λ = 0.1 (across all levels of load and delay, data set G1). The models generating the data are otherwise identical to those described in section 4.1 and we generated 50 trials per condition for each of 10 subjects. For data set G0 model MG0 was favoured in 9/10 subjects, MG1 was favoured in 1/10 subjects, and MG4 in 0/10 subjects. The average (over subjects) log Bayes Factors were 3.42 in favour of MG0 versus MG1, and 5.75 in favour of MG0 versus MG4. For data set G1 model MG0 was favoured in 0/10 subjects, MG1 was favoured in 10/10 subjects, and MG4 in 0/10 subjects. The average log Bayes Factors were 151.3 in favour of MG1 versus MG0, and 4.88 in favour of MG1 versus MG4. Fig 15 shows that model MG1 provided accurate estimates of guess rates across both data sets.

For the location-report task model selection favoured the inclusion of guessing terms. Model MG0 was favoured in 1/10 subjects, MG1 was favoured in 4/10 subjects, and MG4 in 5/10 subjects. The average log Bayes Factors were 4.4 in favour of MG1 versus MG0, and 2.1 in favour of MG4 versus MG1. Thus, the best model is the same as that described earlier with model parameters as shown in Figs 12 and 13.

For the color-report task Model MG0 was favoured in 7/90 subjects, MG1 was favoured in 46/90 subjects, and MG4 in 37/90 subjects. The average log Bayes Factors were 91.4 in favour of MG1 versus MG0, and 0.74 in favour of MG4 versus MG1. Thus, the best model is one with at least one guessing parameter.

In terms of model parameters, the relative strength of the attractor at low memory loads was estimated to be weaker for models without guessing terms (location task: 172:7 without guessing versus 197 with, color task: 48 without guessing, 134 with) indicating that what were previously accounted for as guesses are instead accommodated by weakening the attractor. However, as indicated by the model comparisons, models that included guessing terms provided a more accurate description of the data. We return to this issue in the discussion.

## 4 Discussion

The Stochastic Attractor model of visual working memory proposed by Panichello et al. [12] comprises a Pure-Diffusion variant in which memory traces diffuse away from their initial conditions and a Response-Bias variant in which memory traces are made more robust via attraction to archetypal values. One of the main findings of their work was that empirical data do not support Pure-Diffusion processes—they found clear evidence in favour of “error-correcting” attractor dynamics. In this paper we have proposed a third model variant, the Multi-Item model, in which attractor states correspond to the multiple items to be remembered on a single trial.

Our simulation results indicate that it is possible to identify the three model variants from empirical data. However, the Response Bias model cannot be reliably recovered at the individual subject level unless there is a very large number of trials per subject. The multi-item and pure diffusion model variants, on the other hand, are readily identifiable at both within-subject and group levels.

Across both empirical data sets (colour and location report tasks) we found no evidence for Pure-Diffusion. This is in agreement with Panichello et al. [12]. For the location report task we found clear evidence in favour of the Multi-Item model. For the colour report task we found mixed results. At the group level, the Response-Bias model was favoured. This is in agreement with Panichello et al who also reported a group-level analysis. At the single subject level, however, the Multi-Item model was favoured. Thus, although response biases are clearly manifest at the group level, the attraction of memory traces toward target and non-target items, as captured by the Multi-Item model, is a stronger effect. This analysis was confirmed by a cluster analysis of participant responses which revealed that multiple clusters of response cannot reliably be identified at the single subject level. These findings are also consistent with our simulation results which indicate that the RB model can be reliably identified at the group-level, but without unrealistic sample sizes, not at the subject-level.

A consistent finding across both empirical data sets was that the multi-item model provided the best explanation of the data. A possible concern regarding this and other findings is that the location data set contains only a small number of trials per subject. Nevertheless we can be confident in our findings because the reliability of the results is indicated in a number of ways: (i) the posterior distribution over model parameters for individual model fits (see Eq 18), (ii) Bayes Factors for model comparisons (these naturally become smaller with smaller data sets [51]) and (iii) bootstrap estimates of parameters at the group level (for example, the violin plots in Fig 13 show that as well as guess rates increasing at load 4, the uncertainty in these estimates also increases).

The multi-item model proposes a simple mechanism by which swap-errors arise: memory traces diffuse away from their initial state and are captured by another items’ attractor. We have proposed a new concept, the Swap-Error Curve, defined as the swap error probability as a continuous function of delay time, and have shown how it can be inferred from experimental data (see Figs 11 and 14). This is possible because Stochastic Attractors model the evolution of the recall density as a continuous function of time. The swap error probability at any one time is given by the proportion of probability mass captured by non-target attractors.

In this paper, effects of load are mediated through the diffusion parameters *σ*^2^ that are allowed to vary across load. In previous work Panichello et al [12] have also allowed attractor strength *β* to vary across load, and one of their empirical findings was that load increases both *σ*^2^ and *β* and it was reported that increases in diffusion noise are held in check at higher loads through stronger error-correction. In our simulation work, however, we found that parameter estimates of *σ*^2^ and *β* are highly correlated. Indeed, if we generate data in which only *σ*^2^ is increased across load then we find that both estimates of *σ*^2^ and *β* are increased. Although Panichello et al. report increases in both *σ*^2^ and *β* with load, if we compute the ratio of *β*/*σ*^2^ (using the source data that accompanies Fig 6 in [12]), on average over subjects, this goes down from 15.3 at load 1 to 12.7 at load 3. Thus the relative strength of the attractor reduces. This is in line with our findings on both empirical datasets. Overall, we therefore conclude that it is difficult to identify independent effects of load on diffusion and attractor strength using behavioural data alone and that it is better to make inferences regarding relative attractor strength as a function of load. Further, behavioural data provides evidence that relative attractor strength decreases with load.

### 4.1 Future work

Whilst this paper has shown that a multi-item stochastic attractor model provides a good description of data from two empirical data sets, further work should validate it across a much broader selection of data encompassing a variety sensory features (e.g. orientation and shape as well as colour and location), delay lengths (e.g. long-term as well as short-term memory) and a variety of experimental paradigms (e.g. change detection and cue prioritization as well as continuous report).

The Stochastic Attractor model, proposed by Panichello et al and including the multi-item variant in this paper, considers there to be a decision noise process that is active during the recall period that further diffuses the distribution over memory traces—see Eq 9. However, in future work one might consider letting this diffusion process run as long as the Reaction Time (RT) on each trial, thus making contact with the considerable literature on drift diffusion models of decision making [52] and working memory [53] and use empirical RT data to further constrain parameter estimation. A more mechanistic approach would be to characterise how a cue initiates the recall process (see multi-attribute model in S1 Text) and incorporate this into Bayesian decision theory (see review of Bayesian decision models for WM in Bays et al [22]).

The multi-item model has assumed a Piecewise Sinusoidal (PS) flow function which has the benefit of not having additional parameters to estimate whilst satisfying the main requirement that stable fixed points are created for all items to be remembered. However, PS flow functions have three rather specific characteristics; (i) basins of attraction will be asymmetric unless memory items are uniformly spaced, (ii) the size of each half of the basin of attraction (clockwise or anti-clockwise) depends on distance between memory items, and (iii) they produce global attractors because traces always experience an attractive force (flow functions are only zero at the stable fixed points). In future work it would therefore be of interest to examine, for example, flow functions that produce local rather than global attractors. One potential form of local attractor is described in S1 Text.

Another possibility here would be to estimate multi-item flow functions from simulations of neural network models that embody attractor dynamics e.g. [18, 34, 35]. A further step would be to characterise how the flows change as a function of neuronal or experimental factors. This views the flow function as a central quantity of interest in working memory research that may help to bridge the gap between cognitive and neuronal models.

We found that, in both the color report and location report tasks, models with guessing terms were strongly preferred and find these results to be reliable as guess rates were veridically estimated when using simulated data. Previous research, however, using Variable Precision [25], Target Confusability Competition [26] and Population Coding [33] models have shown that guessing terms are unnecessary if other aspects of the model are correctly configured. This therefore raises the possibility that the PS flow functions instantiated in the multi-item model are not an accurate characterisation of empirical WM systems. Conceptually, one would expect that local attractor flows would yield the necessary heavier-tailed error densities (than the global attractors specified by PS) and are therefore worthy of further enquiry.

Another direction for future work is to model both the evolution of the reported attribute (as in the current paper) *and* the cued attribute using stochastic attractors. Inference over a latent variable would then bind the two features together at retrieval. This “multiple attribute” approach would provide a formal model of feature binding that could scale up to large numbers of features. This is described mathematically in S1 Text and might explain the empirical finding that swap errors can depend on cue similarity [54, 55].

One could additionally add an extra hierarchical dynamical layer to the model whereby the locations of the stable fixed points themselves evolve according to their own flow function (see S1 Text). This would provide a straightforward way to account for repulsion effects whereby fixed points repel if initially too close [32]. The resulting increase in distinctiveness of memory traces is thought to improve feature binding [56]. If this was embodied in a multi-attribute model, then this improvement due to feature binding would fall out mathematically.

Our results on the colour report task indicate that some effects require characterisation at the group level, e.g. response biases, whilst others are evident at the single-subject level, e.g. attraction to the stable states of multiple items. A third direction for future work is therefore to incorporate all effects into a single model and estimate model parameters using a Hierarchical Bayesian approach [57]. The within-subject Laplace approximation adopted in this paper would fit naturally into such an approach and could make use of the variational mixed effects algorithm [46, 58].

The final direction is to apply the models to datasets from clinical neuroscience, for example [59], to identify basic mechanisms that go awry in clinical populations (e.g. excessive diffusion in the cued attribute or the recall attribute, weak overall error-correction, insufficient attractor repulsion) and to relate these to demographic, pharmacological and other clinical factors.

## Supporting information

S1 Text Supporting text. Includes potential extensions of the Stochastic Attractor Model.
(PDF)
