# Supplementary material for: Stochastic attractor models of visual working memory
Source: PLoS One. 2024 Apr 3;19(4):e0301039. doi: 10.1371/journal.pone.0301039 (PMC10990203; doi:10.1371/journal.pone.0301039)
Supplement: S1 Text — Includes potential extensions of the Stochastic Attractor Model. (PDF) [file pone.0301039.s001.pdf]

Stochastic Attractor Models of Visual Working Memory: Supporting Information

W Penny

School of Psychology,
University East Anglia, UK.

March 15, 2024

1 Local Attractors

Here we define a flow function, $g(x)$, that creates local attractors where basins of attraction have width δ around memory items (a parameter to be estimated). The flow function is created using windowed sinusoids

$$\begin{aligned} g(x) &= \sum_{j=1}^{m} w(x, c_j) s(x, c_j) \\ w(x, c_j) &= H(|x - c_j| < \delta/2) \\ s(x, c_j) &= -\sin(2\pi(x - c_j)/\delta) \end{aligned} \quad (1)$$

where c_j is the color of the jth item (or location/orientation depending on experimental paradigm), and $H(a)$ is the Heaviside function (1 for a > 0, 0 otherwise). An example of such a flow function is shown in Fig 1.

2 Multiple Attribute Model

In the main text we have described a stochastic attractor model of a single attribute. Here we describe a generic approach in which each item to be remembered comprises a set of attributes. That is, where $y = \{x_1, x_2, ...x_P\}$. For example, for $P = 3$ we may have object location, size and identity. This model assumes that memory traces evolve independently (see last paragraph below for further comment) for each attribute

$$p(y|z = j) = \prod_{i=1}^{P} p(x_i|z = j) \quad (2)$$

and the density of the ith attribute evolves according to stochastic attractor dynamics

$$p(x_i|z = j) = \exp[\sigma_{ri} D] \exp[M_i \tau] \exp[\sigma_{ei} D] \delta(x_i - x_{ij})$$

where x_{ij} is the encoded value of attribute i for the jth item, σ_{ei} is encoding noise, M_i reflects attractor dynamics, σ_{ri} is read-out noise, and τ is the delay length (as in Eq **??** in the main text).

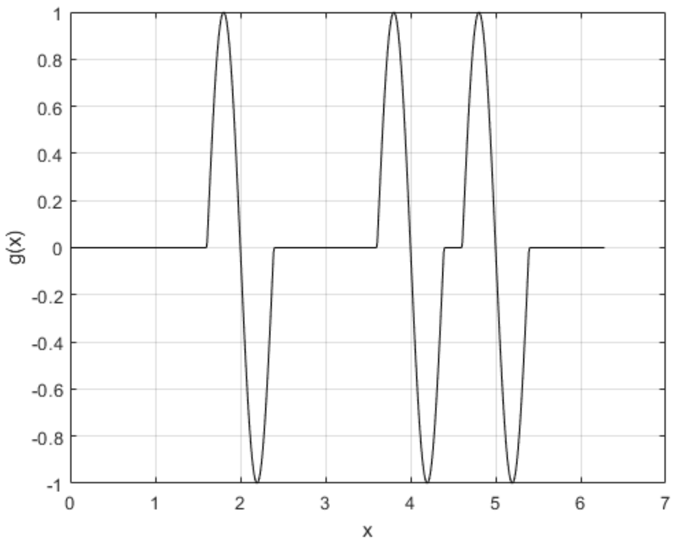

Figure 1: **Local Attractor Flow Function** *The Piecewise Sinusoidal functions used in the current paper instantiate global attractors because memory traces always experience an attractive force (flow functions are only zero at the stable fixed points - See Fig 2 in the main paper). An alternative choice would be a Local Attractor, as shown in the figure, and defined using windowed sinusoids. This contains regions of zero flow where traces are subject only to diffusive forces.*

A new index $z = j$ is assigned to each new stimulus array, as in e.g. Hippocampal indexing theory [1]. The recall density over x_i given *single* cue x_k is computed as follows. First, the cue information is used to update the distribution over indexes

$$\begin{aligned} p(z = j|x_k) &= \frac{p(x_k|z = j)p(z = j)}{p(x_k)} \qquad (3) \\ p(x_k) &= \sum_{j'=0}^{J} p(x_k|z = j')p(z = j') \end{aligned}$$

Second, the updated distribution over indexes is used to compute the recall density

$$p(x_i|x_k) = \sum_{j=0}^{J} p(x_i|z = j)p(z = j|x_k) \qquad (4)$$

The overall model structure is equivalent to a Mixture Model [2] but with multiple independent attributes (we would call this a mixture of stochastic attractors). The above equations derive from Bayes rule and the prior over indexes, $p(z = j)$, could be taken as uniform or have some temporal structure [3]. Because the recall density depends on the cue, this should explain the empirical finding that swap errors can depend on cue similarity (as well as similarity of the recalled attribute) [4, 5]. In this view, these two types of swap error have different causes - diffusion in the cue attribute and diffusion in the recall attribute.

In terms of a potential mapping to neuroanatomy, we envisage that the attribute memory traces are instantiated in the relevant cortical areas and that these can be indexed via discrete latent

variables (or ”tokens/indexes”) instantiated in e.g medial temporal lobe [1, 6, 7, 8] or claustrum [9].

Equation 3 describes how cue information is used to update the distribution over indexes, and may reflect cortical-to-hippocampal signalling and hippocampal normalisation (e.g. via recurrent competition). Equation 4 shows how the index distribution is used to create the predictive density over the recall attribute, and may reflect top-down signalling from hippocampus to the relevant cortical area. In this view ”feature-binding” is instantiated in both bottom-up and top-down signalling.

A reasonable critique of the independence assumption in Eq 2 is that not all cortical attributes evolve independently. For example, there is evidence that location and orientation are coded as a bivariate quantity, a low-level coding arising from orientation selective neurons distributed across the visual field [10]. This could however be accommodated by treating location and orientation as a single bivariate attribute. For higher level attributes e.g. object identity, an assumption of independence between e.g. location and identity (given z) seems more plausible.

If there are multiple cues $m_k = \{x_k, ...x_K\}$, the corresponding feature binding computations are

$$\begin{aligned} p(z=j|m_k) &= \frac{p(z=j)\prod_k p(x_k|z=j)}{p(m_k)} \\ p(m_k) &= \sum_{j'=0}^{J} p(z=j') \prod_k p(x_k|z=j') \end{aligned} \tag{5}$$

and

$$p(x_i|m_k) = \sum_{j=0}^{J} p(x_i|z=j)p(z=j|m_k) \tag{6}$$

3 Hierarchical Dynamics

This model incorporates two levels of dynamics

$$\begin{aligned} d\mu_i &= \alpha f_i(\mu_i) \\ dx_i &= \beta g(x_i, \mu_i)dt + \sigma dw \end{aligned} \tag{7}$$

where, at the top level, the stable fixed points repel each other according to deterministic flow function f_i and, at the bottom level, memory traces follow stochastic attractor dynamics with stable states μ_i (as in the current paper). The top-level flow function could be instantiated using piecewise sinusoids (as in the current paper) but with e.g. *unstable* fixed points located midway between item values for repulsive dynamics. The strength of the different types of effect would be reflected in the parameter values α and β.

4 Integrated Model

In an integrated model we could test for both response-bias and multi-item dynamics

$$dx = \beta_r g_r(x)dt + \beta_m g_m(x)dt + \sigma dw \tag{8}$$

We could also augment this to include other effects, for example, of previous trials (as in [11]) giving

$$dx = \sum_i \beta_i g_i(x)dt + \sigma dw \tag{9}$$

where $g_i(x)$ is the flow function associated with the ith source of dynamical effects (response bias, multi-item, previous trial), and β_i is the corresponding strength.

References

[1] T Teyler and J Rudy. The hippocampal indexing theory and episodic memory: Updating the index. *Hippocampus*, 17:1158–69, 2007.

[2] C.M. Bishop. *Pattern Recognition and Machine Learning*. Springer, New York, 2006.

[3] S Gershman, H Monfils, K Norman, and Y Niv. The computational nature of memory modification. *eLife*, 6:e23763, 2017.

[4] P Bays. Evaluating and excluding swap errors in analogue tests of working memory. *Scientific Reports*, 6:19203, 2016.

[5] J McMaster, I Tomic, S Schneegans, and P Bays. Swap errors in visual working memory are fully explained by cue-feature variability. *Cognitive Psychology*, 137:101493, 2022.

[6] A Borders, C Ranganath, and A Yonelinas. The hippocampus supports high-precision binding in visual working memory. *Hippocampus*, pages 1–14, 2021.

[7] N Rose. The dynamic-processing model of working memory. *Current Directions in Psychological Science*, 29:378–387, 2020.

[8] A Beukers, T Buschman, J Cohen, and K Norman. Is activity silent working memory simply episodic memory? *Trends in Cognitive Sciences*, 25:284–293, 2021.

[9] S Hedayati, R O'Donnell, and B Wyble. A model of working memory for latent representations. *Nature Human Behaviour*, 2022.

[10] S Schneegans and P Bays. Neural architecture for feature binding in visual working memory. *The Journal of Neuroscience*, 37:3913–3925, 2017.

[11] K Wimmer, D Nykamp, C Constantinidis, and A Compte. Bump attractor dynamics in prefrontal cortex explains behavioural precision in spatial working memory. *Nature Neuroscience*, 17:431–439, 2014.
